# Contrast-enhanced ultrasonography (CEUS) in the management of pediatric renal injuries: where are we now?

**DOI:** 10.1007/s40477-025-01011-0

**Published:** 2025-03-22

**Authors:** Dacia Di Renzo, Cristina Gentile, Antonello Persico, Giuseppe Lauriti, Francesco Chiarelli, Gabriele Lisi

**Affiliations:** 1 Ultrasound Service of Pediatric Surgery, “Spirito Santo” Hospital of Pescara, Pescara, Italy; 2 Department of Pediatrics of “G. d’Annunzio”, University of Chieti-Pescara, Chieti - Pescara, Italy; 3 Present Address: Pediatric Surgery of “G. d’Annunzio” University of Chieti-Pescara, Chieti - Pescara, Italy

**Keywords:** Contrast-enhanced ultrasonography, CEUS, Renal trauma, Kidney, Pediatric, Pediatric ultrasonography

## Abstract

**Purpose:**

Experience with CEUS in management of kidney post-traumatic injuries is limited, especially in pediatric age. This paper aimed to identify: clinical settings in which CEUS could be used as first diagnostic tool, skipping CT; CEUS ability to detect complications during non-operative management (NOM); and CEUS role in patients with collecting system injuries.

**Methods:**

Patients with renal trauma admitted between 2003 and 2023 were enrolled in a retrospective study. At T0, CT was performed in case of high-energy trauma, CT or CEUS in case of low-energy or/and localized trauma. CEUS was used during follow up (FU) in case of suspected complications and to follow healing of the lesions and urinomas.

**Results:**

Among 22 patients included, at T0 20/22 performed CT, 1/22 CEUS and 1/22 baseline US. During NOM CEUS was necessary: in early FU to rule out complications in 3/22 (1 anemization and 2 hematuria); in middle FU in 14/22 to authorize mobilization/discharge and monitor urinomas; in outpatient setting in 2/22, to authorize return to sport activities. Overall, a collecting system injury was detected in 6 patients by CT and in 1 by CEUS. In 3/7 a perirenal urinoma developed. All were monitored with CEUS or baseline US.

**Conclusions:**

CEUS is useful as first imaging study in low-energy and localized trauma, but confidence with CEUS is still to be improved and spread, to replace CT in selected cases. CEUS is valuable for detecting complications, avoiding repeat CT in most of cases. In expert hands CEUS can identify and monitor leakage indirectly.

**Graphical abstract:**

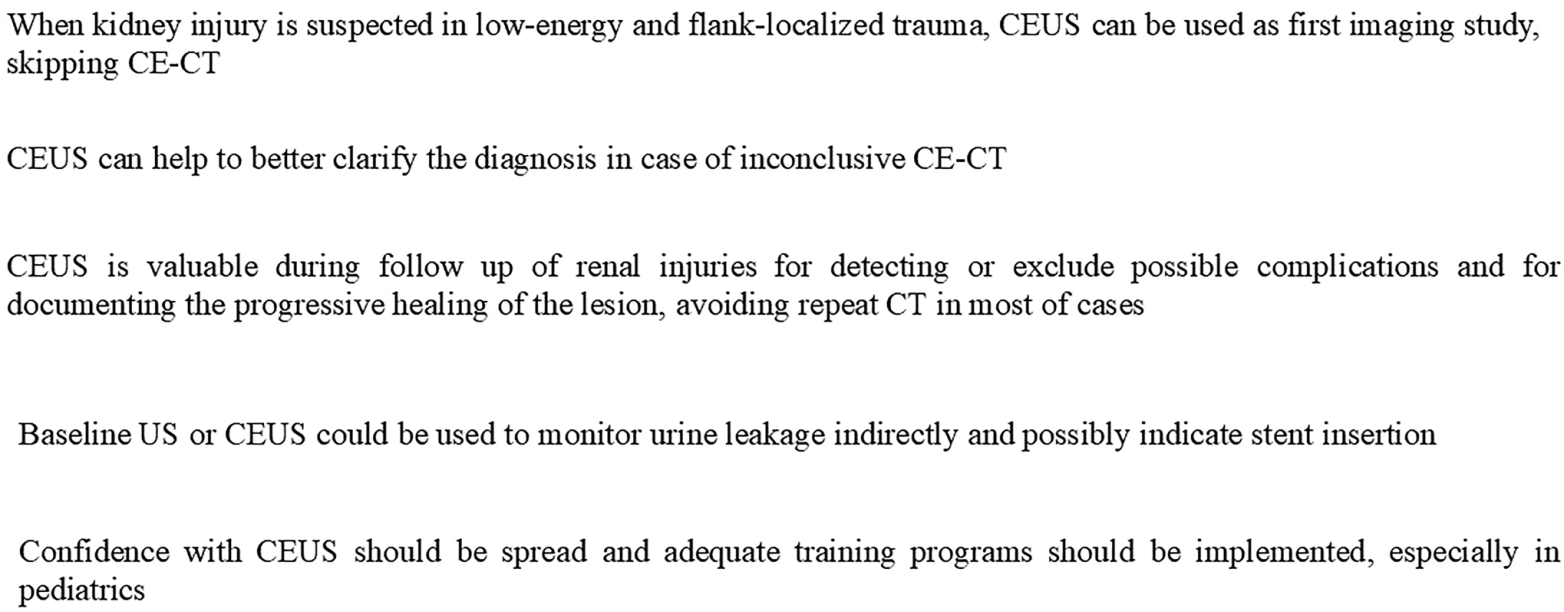

## Introduction

Trauma remain the major cause of morbidity and mortality in pediatric age, with abdominal involvement rate up to 25% [1]. Renal trauma accounts for 10–20% of all pediatric cases of blunt abdominal trauma [2]. Contrast-Enhanced Computed Tomography (CE-CT), with delayed imaging to study urinary tract, is the gold standard imaging study for the diagnosis and the evaluation of traumatic renal lesions, particularly to detect possible urinary leakage due to collecting system injury [3].

In the last decades, Contrast-Enhanced Ultrasonography (CEUS) has been used as an alternative to CE-CT to study patients with abdominal traumatism in certain scenarios, especially in those children who have sustained low-energy and/or localized blunt abdominal trauma [4–6]. Several Authors have reported their experience in the management of splenic and hepatic post-traumatic injuries, using CEUS either to detect the lesions and/or to follow their healing [7–12]. On the contrary, experience with CEUS in management of kidney post-traumatic injuries is more limited, especially in pediatric age, with few cases included in heterogeneous populations [9, 11, 13–16] or as preliminary reports [17]. The most remarked issue is about CEUS inability to directly detect lesions of the collecting system, due to the lack of urinary excretion of USCAs.

In this paper we report our experience with CEUS in the management of kidney injuries in pediatric age, discussing consolidated and potential application of CEUS. The main aim of the study is to investigate the role of CEUS in the diagnosis and follow up (FU) of post-traumatic renal injuries in children. In detail, we aimed: (i) to identify the clinical settings in which CEUS could be used as first diagnostic tool, skipping CT; (ii) to identify CEUS ability to detect potential complications during non-operative management (NOM); (iii) to identify CEUS role in patients with collecting system injuries, subjected to non-operative or mini-invasive management.

## Materials and methods

In the present study, after approval, we retrospectively reviewed the medical records of all patients admitted for kidney trauma at the Pediatric Surgery Unit of our hospital, during a twenty-year period, from 2003 to 2023. Data regarding age, sex, trauma mechanism, type and degree of injury, possible collecting system injury, associated extra-renal lesions, diagnostic tests performed at the admission (T0) and during FU, and therapeutic interventions (both medical and/or surgical) were collected. All injuries were classified according to the revised American Association for the Surgery of Trauma (AAST) organ Injury Scoring Scales (IIS) [18] and treated operatively or not according to the relative risk class [19].

CE-CT was performed at T0 in all hemodynamically stable patients undergoing high-energy abdominal blunt trauma. In patients with low-energy trauma, CE-CT or CEUS was performed at T0, based on trauma mechanism, clinical conditions, baseline ultrasonography (US) results, and availability of the physician who performs CEUS exams. We underline that the majority of these patients were admitted in our Unit with a CE-CT study already performed in the emergency department. NOM included closely vital signs and laboratory test monitoring, supportive care, re-imaging if deterioration occurred.

During NOM, CEUS was performed in case of suspected complications (symptoms, laboratory tests alterations, etc.) to identify possible persistent or delayed bleeding, arteriovenous fistula, pseudoaneurysm, and perinephric urinoma or abscess; moreover, CEUS was performed to monitor the evolution of the renal injuries as well as to authorize the mobility of the patient and the return to sport activities.

All CEUS were performed by the same operator, at the patient's bedside or in outpatient setting for late FU examinations. The Second-generation Ultrasounds Contrast Agent (USCA) SonoVue^®^ (Bracco, Milan, Italy), currently approved in Europe, and dedicated software were used for each exam. According to the latest FDA indications (https://www.fda.gov/media/98508/download), a single dose of 0.03 mL/ Kg up to a maximum of 2.4 mL per injection, was intravenously administered, based on the patient's weight. A peripheral vein was used and the bolus of SonoVue^®^ was followed by 10 mL of saline solution. Due to the current pharmaceutical regulation, SonoVue^®^ was used “off-label”; therefore, an informed consent was collected for each patient prior to examination. All measures to identify and treat a possible adverse reaction have been taken during each exam. CEUS execution was avoided in the presence of contraindications, such as history of known hypersensitivity to contrast agents, right-to-left-shunt, severe pulmonary hypertension, systemic hypertension, and uncertain pregnancy status.

## Results

Between October 2003 and October 2023, a total of 84 patients were admitted for blunt abdominal trauma with detection of parenchymal lesions to the Pediatric Surgery Unit of “Santo Spirito” Hospital of Pescara, Italy; among these, 22 patients (26%) reported a renal injury. Table 1 shows the main demographic characteristics of included patients and traumatic mechanism.

**Table 1:** Characteristics of included patients

Patient	Age	Sex	Mechanism of injury	AAST Grade	Side	Associated intra-abdominal lesions	Hospital stay (days)
1	12	M	Fall from height	III	Right	–	8
2	16	M	Road accident	III	Left	Spleen	9
3	16	F	Road accident	II	Right	–	7
4	10	F	Fall from bicycle	II	Right	Liver	9
5	4	M	Fall from height	IV	Left	Spleen	13
6	15	M	Road accident	IV	Right	Liver, adrenal gland	11
7	13	M	Abdominal crush	III	Left	Spleen	18
8	13	M	Fall from bicycle	I	Left	Spleen	12
9	11	F	Road accident	IV	Left	Spleen	17
10	12	M	Abdominal crush	IV	Right	–	12
11	16	M	Sport trauma	IV	Left	Spleen	9
12	15	F	Violent contact	I	Left	Spleen	15
13	8	M	Fall from height	IV	Right	–	8
14	11	F	Fall from horse	I	Right	Liver	13
15	11	M	Fall from stairs	I	Right	Liver, adrenal gland	7
16	16	F	Road accident	I	Left	Spleen	24
17	6	M	Fall from stairs	III	Left	–	14
18	14	M	Road accident	I	Left	–	3
19	16	M	Knife wound	III	Right	Liver	13
20	15	M	Fall from bicycle	IV	Right	–	25
21	16	F	Road accident	IV	Left	Spleen	8
22	7	F	Fall from stairs	IV	Left	–	7

Nine-teen of 22 patients underwent abdominal or total-body CE-CT at admission (T0); in 1/22 CE-CT was performed 3 days after trauma, because at T0 a baseline US excluded parenchymal lesions and the patient was discharged from ER; 3 days later he returned because of vomiting and flank pain and a CE-CT revealed a renal injury; in 1/22 CEUS was performed at T0, followed by a CE-CT; 1/22 performed only baseline US at T0, detecting a renal contusion. Unexpectedly, in 1 patient whose CT performed at T0 was negative for renal lesions, a baseline US performed the 2nd day post-admission for microscopic hematuria, suspected a renal lesion, confirmed by CEUS.

According to AAST classification of renal injuries, we observed grade IV lesions in 9/22 patients, grade III in 5/22, grade II in 2/22 and grade I in 6/22. No grade V lesions were detected in our cohort. An isolated renal lesion was detected in 8/22 patients; the remaining 14 cases had combined lesions: 9 combined splenic and kidney injuries, 3 combined hepatic and kidney, 2 combined kidney, liver and adrenal. The left side was the most frequently involved (12/22 patients, 54.5%). Associated extra-abdominal lesions were present in 10/22 patients: 5 had bone fractures, 4 thoracic involvements with pneumothorax, pulmonary contusion, or pleural effusion, 4 head or cervical trauma.

A collecting system injury was highlighted in 7 patients: in 6 of these the lesion was detected by CE-CT while in 1 patient CEUS was diagnostic. In three of these 7 patients with collecting system injury, a perirenal urinary extravasation (urinoma) developed; in the remaining 4, no significant urinary extravasation was detected. Microscopic hematuria, defined as three or more red blood cells per high-power field, was found in 12/22 patients.

Out of the total, 15 patients performed at least one CEUS (Table 2). In detail, 10/15 patients underwent one CEUS, 5/15 two CEUS, for a total of 20 CEUS performed. The timing for follow up CEUS was “patient-tailored”, according to the clinical conditions and degree of renal lesion.

One/15 patients underwent CEUS at T0; in 3/15 CEUS was performed in the early FU (between the 2nd and 5th day post trauma) to detect possible complications: in 1 case for new onset anemia and persisting important hematuria, in 2 cases for onset of hematuria, not present before (in 1 of these 2, a caliceal lesion not detected at CT was found). Fourteen CEUS were performed in the middle-late FU, to assess the evolution of the lesion and to allow patient mobilization or discharge; in 2 cases, an additional CEUS was performed after discharge, in outpatient setting, to document complete healing of the lesion. Only 1/15 patients repeated CE-CT during FU (the indication was persistent vomiting in a patient with important brain contusions).

None of the patients had contraindications to CEUS and no adverse reaction related to USCAs administration was reported in our cohort. Except urinomas, no other complications including arteriovenous fistula, pseudoaneurysm, or perinephric abscess has been encountered.

Non-operative management (NOM) was successful in most of patients (13/15). In 2 cases, due to the presence of persisting urinoma, a cystoscopy with a ureteral stent insertion was necessary, in II e IV-day post-trauma. In 1 of these 2 patients, due to persistent bleeding, an arterial embolization was contextually performed. Blood transfusion was required in 2 patients because of acute onset anemia. However, in both cases, a splenic lesion was associated.

Median hospital stay was 13.3 days (range 7–25 days).

## Discussion

Kidneys are involved in about 10–20% of blunt abdominal trauma cases [2], with well-recognized predisposing factors, especially in pediatric age [20–22]. High-velocity deceleration represents the most common mechanism for renal injuries (90% of cases), whereas penetrating traumas, such as gunshot and stab wounds, are rare (1.4–3.3%) [23].

Until 20 years ago, early operative management was considered an imperative choice, especially in pediatric population, to reduce morbidity and mortality. However, in last decades, a great number of studies reported encouraging data on the efficacy and safety of NOM, even in children with high-grade renal injuries [24, 25]. In a recent systematic review, LeeVan et al. have reported a success rate of NOM in about 80% of grade IV-injuries, with a renal salvage rate greater than 95% in the majority of the included studies [26]. Thus, as in adults, even in children with hemodynamically stable kidney trauma, NOM has become the first therapeutic option.

This changing in management protocols has increased the need for more accurate diagnostic tools, to early identify renal lesions, correctly establish their grade and avoid complications. As CE-CT with delayed imaging of urinary tract is still considered the gold standard technique for the diagnosis and the evaluation of traumatic renal lesions in hemodynamically stable patients [19], its indiscriminate use in pediatric population could lead to an excessive exposure to ionizing radiation and to possible adverse reactions to iodinate contrast agents. The Royal College of Radiology recently released the “Major Pediatric Trauma Radiology Guidance” [27], in which it was stated that in children there is increased risk of ionizing radiation, so the ALARP (as low as reasonably practicable) principle should be adhered to. It is also remarked that CT is helpful in the pediatric trauma setting but is not mandatory and should be tailored to the patient. While the use of focused abdominal US in trauma and unenhanced US is not appropriate and can provide false reassurance, CEUS could be considered appropriate in cases once discussed with an expert.

The introduction USCAs in medical practice has led to a significant increase in US diagnostic accuracy [15, 28]. In a recent prospective study on 18 pediatric blunt abdominal trauma patients, an improvement of US-sensitivity from 45 to 86% and US-specificity from 96.4% to 98.6% using CEUS has been reported [16]. Moreover, many studies reported a similar sensitivity between CEUS and CE-CT in the identification and characterization of intra-abdominal injuries, including kidney trauma. In a recent meta-analysis, Zhang et al. have reported a sensitivity of CEUS of 98.1%, with an overall false-positive rate of 1.8% compared to CT [10]. These results confirm what Menichini and coll. have already reported in a previous study [9]. Furthermore, CEUS shows relevant advantages compared to CE-CT, especially in pediatric population. They include an extreme safety profile even in patients with renal function impairment [29], a non-significant rate of adverse reactions to USCAs [30–32], and the possibility to be performed at the patient's bedside. USCAs consist of gas microbubbles, perfluorocarbon or sulfur hexafluoride, surrounded by a shell protein, lipid or polymer [33]. The second-generation USCA Sonovue^®^ (Bracco, Milan, Italy) is currently approved in Europe for characterizing focal liver lesions and for use during echocardiography in pediatrics [34]. Renal enhancement is characterized by two different vascular phases: the “cortical” phase, that occurs immediately after intravenous administration and main renal artery enhancement (10–15 s after injection); the “medullary” phase which begins after 40 s and lasts about 120 s. Since the contrast is not metabolized by the kidneys, no excretory phase is observed. Thus, any alteration of normal architecture and perfusion of the kidneys can be assessed by CEUS. In detail, during the post-contrast venous phase, any acute injury appears as a non-enhancing defect with a sharp distinction from the normal vascular tissue. Any laceration appears as a linear, branching defect that is perpendicular to the overlying capsule associated with the capsular breach. Lenticular non-enhancing subcapsular hematoma can also be seen. Active extravasation of contrast can also be appreciated on CEUS as microbubble extravasation into renal or perirenal space. Complete vascular injury due to pedicle avulsion is characterized by the complete absence of renal perfusion. However, due to lack of urinary excretion of USCAs, CEUS in unable to directly detect lesions of the collecting system [35].

Although the role of CEUS in the management of intra-abdominal injuries has been widely demonstrated, specific data on its value in diagnosis and follow up of post-traumatic renal injuries are still scarce. Moreover, most of the studies published up to now have included few kidney lesions in mixed adult-pediatric populations [9, 11, 13–16].

Recently, preliminary results on the use of CEUS in renal trauma in a small number of exclusively pediatric patients has been published by Bowen et al. [17]. This retrospective study was conducted in a small cohort of 7 patients: CEUS was used in comparison to CECT in 4 of these 7 patients as an initial evaluation or during short-term follow-up (2 days after trauma). CEUS alone was used in 1 patient as an initial evaluation and in 2 cases at follow-up. This study highlights the value of CEUS in minor trauma with low suspicion of renal injury—where CE-CT can be skipped—or in cases of inconclusive CE-CT, especially in those children referred from other hospitals, where CEUS can be performed, to prevent a repeated CE-CT. It also highlights the role of CEUS at FU, due to its capacity to closely monitor the evolution of lesions through the visualization of perfusion in injured areas and the estimation of parenchymal loss, hence avoiding the ionizing radiation exposure and minimizing the risk of adverse reactions to iodinate contrast agents. Moreover, in contrast to previous studies that highlight the poor ability to detect active bleeding [9], Bowen et al. demonstrate that CEUS could clearly detect contrast pooling around the kidney suggestive of an active bleeding.

To our knowledge, there are no other studies specifically focusing on CEUS role in kidney trauma in exclusively pediatric populations. In our study we report our experience with CEUS in management of pediatric renal injuries with the purpose to identify: clinical settings in which CEUS could be used as first diagnostic tool, skipping CT; CEUS ability to detect potential complications during NOM; and CEUS role in patients with collecting system injuries. According to traumatic mechanism, clinical conditions at admission and internal protocols, 20/22 patients of our cohort underwent CE-CT at T0. Only in 1 case CEUS was performed at T0 but it was integrated by a CE-CT subsequently performed, because it was one of the first CEUS examination performed and we did not feel enough confident. In 1 case CEUS was used only during FU of a suffusion detected by baseline US. This delineates that at our hospital, that is a non-pediatric one, most of abdominal traumas, even if low-energy and localized, are approached with CE-CT. This can be partly explained by the fact that the first evaluation and exams are done in the emergency department and by lack of expertise with CEUS by our Radiologists. Moreover, many patients were firstly studied with CE-CT in peripheral Hospitals and then referred to our Unit. Therefore, we had 8 isolated kidney lesions due to localized trauma, without head or chest involvement, in patients with good general conditions, that could hypothetically be studied at T0 with CEUS.

Thus, given the operator-dependence of CEUS, adequate training programs and a careful selection of cases are required. Including CEUS in diagnostic algorithms, especially in the pediatric population, could allow to a substantial reduction in CE-CT related risks. The possibility to be performed in case of renal function impairment at the patient’s bedside could led also to an acceleration and facilitation of the entire diagnostic process, especially in emergency settings.

Similarly to what has been reported about the diagnostic role of CEUS in renal trauma, data on the role of this imaging tool in FU and its proper timing are still lacking in the literature. In a previous study, we have defined the diagnostic value of CEUS during FU of children with blunt abdominal trauma, even if with few specific data regarding renal injuries [11]. Similarly, Bowen et al., did not provide precise indications on the correct timing [17]. We think that FU timing should be "patient-tailored", based on AAST grade at T0, clinical evolution of the patient, and baseline US and laboratory checks. In the first 72 h post traumas CEUS can be useful in case of anemia or clinical signs of bleeding or hematuria. In our cohort, 1 patient performed CEUS in early-FU, because of new onset hematuria. In this patient, with T0-CE-CT negative for renal lesions, CEUS highlighted a collecting system injury and allowed us to "re-stadiate" the lesion, avoiding a repeated CE-CT.

After the first 72 h post trauma, CEUS can be performed to evaluate the progressive healing of the lesions and to prescribe mobilization and/or discharge. In high-grade lesions, a late follow up CEUS can be required. In our cohort, CEUS at 1-month post-trauma was performed in 2 cases, both after discharge, in outpatient setting.

Thus, even in the FU-contest, CEUS shows numerous advantages: the possibility to perform targeted checks during the hospitalization, especially in case of clinical changes or in high-grade renal lesions, when a prolonged hospitalization is required or in an outpatient setting. Moreover, in a FU setting, the problem regarding the low panoramic view is in part overcome, as the lack of urgency and knowledge of the lesion site allows for the choice of the most appropriate acoustic window. In addition, the lack of ionizing radiation allows a prolonged observation, if necessary, to obtain a detailed assessment of the lesion.

One of the main issue regarding CEUS in the management of traumas of the kidney remains the low capability to study the collecting system involvement and relative complications, such as extravasation of urine and/or urinomas, because of lack of microbubbles urinary excretion [40]. Although has been demonstrated that up to two-thirds of urinomas resolve spontaneously [36], urine leak is often associated with a prolonged hospitalization, increased risk of complications and higher operative management rate [26]. However, although CEUS is not able to directly detect urine extravasation, in our experience a urinoma can be followed with baseline US and, in case of a growing perirenal fluid collection, CEUS allows to exclude urine leak indirectly, as its enhancement indicates the hematic nature of the extravasation while the lack of enhancement can suggest the urinary origin.

Our study has some limitations. First, its retrospective design. Secondly, all CEUS were performed by the same operator who is appropriately trained but not blinded to prior CE-CT results, with a consequent bias in the examination of the kidney. Finally, the absence of a direct comparison between CE-CT and CEUS images during FU, justified by the ALARP principle.

## Conclusions

Our experience confirms CEUS as a useful, handy and safe imaging tool in the management of pediatric renal injuries. During the diagnostic phase it could be considered as first imaging technique, skipping CE-CT, only in selected cases, however adequate expertise is mandatory. CEUS could also help to better clarify the diagnosis in case of inconclusive CE-CT. During FU, CEUS value is consolidated in ruling out complications and in documenting the progressive healing of the lesion. In expert hands, CEUS can also identify and monitor urine leakage indirectly and possibly indicate stent insertion.

In conclusion, the incorporation of CEUS into pediatric renal trauma flow-charts should be seriously considered; therefore, adequate training programs should be implemented.

## Data Availability

Additional data are available on request from the corresponding author.

## Declarations

### Research involving human and /or animal participants

This study involves human participants under 18 years of age; contrast media was “off label” used and informed consent was signed by all parents/caregivers of children included in the study;
